# Management of subtrochanteric femur fractures: is open reduction associated with poor outcomes?

**DOI:** 10.1007/s00068-021-01834-6

**Published:** 2021-11-26

**Authors:** Michalis Panteli, James Shen Hwa Vun, Robert Michael West, Anthony John Howard, Ippokratis Pountos, Peter Vasilios Giannoudis

**Affiliations:** 1 Academic Department of Trauma and Orthopaedics, School of Medicine, University of Leeds, Clarendon Wing, Level D, Great George Street, Leeds, LS1 3EX West Yorkshire UK; 2 Leeds Institute of Rheumatic and Musculoskeletal Medicine, University of Leeds, Leeds, UK; 3 Leeds Orthopaedic & Trauma Sciences, Leeds General Infirmary, University of Leeds, Leeds, UK; 4 Leeds Institute of Health Sciences, University of Leeds, Leeds, UK; 5 NIHR Leeds Biomedical Research Unit, Chapel Allerton Hospital, Leeds, UK

**Keywords:** Subtrochanteric, Open reduction, Cerclage wiring, Complications, Infection, Non-union

## Abstract

**Purpose:**

The aim of this study was to identify factors associated with the need for open reduction in subtrochanteric femoral fractures and investigate the effect of cerclage wiring compared to open reduction alone, on the development of complications, especially infection and non-union.

**Methods:**

All consecutive patients with a fracture involving the subtrochanteric region were retrospectively identified, over an 8-year period. Data documented and analysed included patient demographics, fracture characteristics, patient comorbidities, time to fracture union and development of complications.

**Results:**

A total of 512 patients met the inclusion criteria (523 fractures). Open reduction was performed in 48% (247) of the fractures. Following matching and regression analysis, we identified diaphyseal extension of the fracture to be associated with an open reduction (OR: 2.30; 95% CI 1.45–3.65; *p* < 0.001). Open reduction was also associated with an increased risk of superficial infection (OR: 7.88; 95% CI 1.63–38.16; *p* = 0.010), transfusion within 48 h following surgery (OR: 2.44; 95% CI 1.96–4.87; *p* < 0.001) and a prolonged surgical time (OR: 3.09; 95% CI 1.96–4.87; *p* < 0.001). The risk of non-union, deep infection and overall mortality was not increased with open reduction. The use of cerclage wires [50 out of 201 fractures (24.9%) treated with an open reduction] to achieve anatomical reduction as compared to open reduction alone significantly reduced the risk of non-union (OR: 0.20; 95% CI 0.06–0.74; *p* = 0.015).

**Conclusion:**

Open reduction of subtrochanteric fractures is not associated with an increased risk of deep infection and non-union, even though it is associated with an increased risk of superficial infection, prolonged surgical time and transfusion. The use of cerclage wire is associated with reduced risk of non-union with little evidence of an increase in complications.

**Level of evidence:**

III.

## Introduction

Subtrochanteric fractures are defined as those encountered between the inferior border of lesser trochanter and 5 cm distal to it [1]. They generally account for 5–34% of all proximal femoral fractures [2–4], and the ‘gold standard’ of their treatment is intramedullary (IM) nailing. Their management can be very challenging because of their unique biomechanical and anatomical features (moderate vascularity and high concentration of biomechanical stresses) [5–9]. Furthermore, the multiple deforming forces acting on the fracture fragments make their reduction difficult. Consequently, it is not surprising that a 4.7% reoperation rate has been reported related to fracture non-union and metalwork failure [3, 10].

When closed reduction fails to improve fracture alignment, open reduction has been recommended [11–13]. However, open reduction has been associated with such complications as infection, non-union, prolonged surgical time and blood loss requiring blood transfusion [11–13]. Interestingly, published evidence remains conflicting, with some studies reporting an increased incidence of non-union and infection [14, 15], whilst other studies report no difference with regard to non-union, infection, transfusion rates and fracture vascularity [12, 13]. Overall, there appears to be lack of strong evidence comparing the outcomes of open versus closed reduction in the management of subtrochanteric femoral fractures treated with cephalomedullary nailing.

Therefore, the aim of our study was to report on the factors associated with the need for open reduction and to investigate whether open reduction is associated with a higher risk for complications. Moreover, to assess if cerclage wiring has an impact on the development of fracture non-union. Our hypothesis was that open reduction is associated with an increased risk of infection and non-union, whilst the use of cerclage wires further increases this risk.

## Methods

Following institutional board approval (LTH#2591), we conducted a retrospective analysis of all consecutive eligible patients presenting in a Level I Trauma Centre over a period of eight years (2009–2016). We included all skeletally mature patients presenting with a fracture extending through the subtrochanteric region that was subsequently treated with an IM nail. Exclusion criteria included the following: pathological fractures (secondary to malignancy or atypical fractures), patients receiving prophylactic nailing for tumours (without a fracture) or incomplete fractures and patients who had their primary operation in other institutions.

Information collected included demographics and comorbidities, operation details, complications and outcomes. Fractures were classified according to the Russell Taylor classification system [16, 17]. Radiographic features were also analysed, with all radiographic measurements being performed independently by MP and JV, with any disagreements resolved by the senior author (PVG).

All patients were managed by the multidisciplinary team, under a set protocol. All patients were positioned supine on a traction table, with post in the perineal area and with the contralateral leg held into lithotomy position. Closed reduction was first attempted and if unsuccessful, open reduction was performed. The use of clamp assisted reduction and cerclage wires was left to the discretion of the operating surgeon. Post-operatively, patients followed a strict physiotherapy regime aiming for early mobilisation and were closely monitored for complications. Superficial infection was defined as erythema, pain, swelling, discharge and delayed wound healing, along with raised inflammatory markers in the early post-operative period [18]. Deep infection was defined as infection involving the fascial, muscular layers and beyond, surrounding the metalwork and necessitating further surgical interventions [19]. Superficial infections were generally treated with oral antibiotics, as compared to deep infections that were treated with a combination of intravenous (IV) antibiotics, wound debridement and removal/revision of implants, as deemed appropriate.

### Statistical analysis

Statistical analysis was performed using the computing environment R (R version 3.6.0) [20]. Basic demographic data were presented as count (percentage) or as mean ± SD. Parametric data were analysed using a Welch unpaired independent t-test, whilst count data were analysed using a Pearson’s chi square test. A *p* value of < 0.05 was considered as significant. To reduce the risk of bias from patient or injury characteristics, the groups were randomly matched {smatch function provided by Lewer D [21]} by terms of age (± 5 years), gender and mechanism of injury (low or high energy). The matching ratio was 1 and matching was repeated using the first five seeding combinations to ensure no significant difference was observed in the outcomes of each matching combination. A simple logistic regression model was used for the initial analysis, to identify potential unadjusted associations with open reduction. Then by removing covariates in a stepwise fashion according to their likelihood-ratio chi-square *p* value, we developed a revised adjusted model of multiple logistic regression to investigate associations with open reduction. The same methods were used to investigate associations with cerclage wiring.

### Statistical power

When occurrence of characteristics is close to 50%, the study can detect with 80% power (*α* = 0.05) a difference in prevalence of 12.1%, whereas when occurrence of characteristics is close to 10%, the study can detect with 80% power (*α* = 0.05) a difference in prevalence of 8.8%. We, therefore, look for differences in the occurrence of characteristics which are more than 10%.

## Results

### Demographics, mechanism of injury and operative characteristics

A total of 512 patients (523 fractures; 205 males) fulfilled the inclusion criteria. There was a bimodal male, unimodal older female age distribution, whilst the commonest mechanism of injury was low energy injury.

Overall, 48% of subtrochanteric fractures (247 fractures) required an open reduction to achieve satisfactory reduction and fixation. Matching was then performed to account for the effect of age, gender and type of injury. The mean age of the matched open reduction group was 75.59 ± 17.93 years and 75.19 ± 17.70 years in the closed reduction group (Table 1).

**Table 1:** Table presenting the demographics/characteristics of patients having a subtrochanteric fracture treated with a long cephalomedullary nail, stratified according to open reduction

Demographics	All patients	Closed reduction	Open reduction
Total number	402	201	201
Bilateral	20 (5.0%)	13 (6.5%)	7 (3.5%)
Age (years)	75.39 (17.78)	75.19 (17.70)	75.59 (17.93)
Gender			
Male	148 (36.8%)	74 (36.8%)	74 (36.8%)
Female	254 (63.2%)	127 (63.2%)	127 (63.2%)

Injury characteristics	All patients	Closed reduction	Open reduction
Mechanism of injury			
Low energy	352 (87.6%)	176 (87.6%)	176 (87.6%)
High energy	50 (12.4%)	25 (12.4%)	25 (12.4%)
Isolated	347 (86.3%)	168 (83.6%)	179 (89.1%)
ISS > 16	20 (4.9%)	11 (5.5%)	9 (4.5%)
Side			
Left	224 (55.7%)	117 (58.2%)	107 (53.2%)
Right	178 (44.3%)	84 (41.8%)	94 (46.8%)
Open fracture	4 (1.0%)	0 (0.0%)	4 (2%)

Medical comorbidities	All patients	Closed reduction	Open reduction
ASA			
1	30 (7.5%)	15 (7.5%)	15 (7.5%)
2	100 (24.9%)	56 (27.9%)	44 (21.9%)
3	203 (50.5%)	96 (47.8%)	107 (53.2%)
4	69 (17.2%)	34 (16.9%)	35 (17.4%)
Charlson comorbidity score	5.35 (2.94)	5.14 (2.91)	5.55 (2.97)
Diabetes	60 (14.9%)	25 (12.4%)	35 (17.4%)
Steroids	15 (3.7%)	8 (4.0%)	7 (3.5%)
Malignancy	90 (22.4%)	45 (22.4%)	45 (22.4%)
Dementia	106 (26.4%)	49 (24.4%)	57 (28.4%)

Osteoporosis	All patients	Closed reduction	Open reduction
Bisphosphonates pre-admission	70 (17.4%)	39 (19.4%)	31 (15.4%)
Bisphosphonates on discharge	100 (24.9%)	55 (28.8%)	45 (23.9%)
Calcium/vitamin D pre-admission	127 (31.6%)	57 (28.4%)	70 (34.8%)
Calcium/vitamin D on discharge	197 (49.0%)	90 (47.1%)	107 (56.9%)
Vitamin D loading on admission	68 (16.9%)	41 (21.5%)	27 (14.4%)
Fragility fractures before	99 (24.6%)	44 (21.9%)	55 (27.5%)
Fragility fractures after	67 (16.7%)	42 (20.9%)	25 (12.5%)
DEXA result			
Normal	3 (8.5%)	1 (4.5%)	2 (15.4%)
Osteopenia	10 (28.6%)	6 (27.3%)	4 (30.8%)
Osteoporosis	22 (62.9%)	15 (68.2%)	7 (53.8%)

Social history	All patients	Closed reduction	Open reduction
Smoking	73 (18.2%)	43 (21.4%)	30 (17.2%)
Alcohol > 10 units/week	71 (17.7%)	37 (18.4%)	34 (16.9%)
Pre-operative mobility			
Independent	192 (47.8%)	99 (49.3%)	93 (46.3%)
Stick(s)/crutch(es)	111 (27.6%)	52 (25.9%)	59 (29.4%)
Frame	82 (20.4%)	40 (19.9%)	42 (20.9%)
Wheelchair/hoisted	17 (4.2%)	10 (5.0%)	7 (3.5%)
Frequent falls	121 (30.1%)	56 (27.9%)	65 (32.3%)

Operation characteristics	All patients	Closed reduction	Open reduction
Operation in less than 48 h	319 (79.4%)	161 (80.1%)	158 (78.6%)
Simultaneous procedures	24 (6.0%)	10 (5.0%)	14 (7.0%)
Use of cerclage wires	50 (12.4%)	0 (0.0%)	50 (24.9%)
Surgical time (min)	110.87 (44.22)	96.58 (35.60)	125.22 (47.36)
Anaesthetic time (min)	48.14 (22.17)	47.97 (21.20)	48.30 (23.16)
Time from induction to recovery (min)	177.70 (48.37)	161.65 (41.19)	193.82 (49.76)
Level of first surgeon			
Registrar	244 (61.2%)	131 (65.5%)	113 (56.8%)
Consultant	155 (38.8%)	69 (34.5%)	86 (43.2%)
Level of senior surgeon present			
Registrar	227 (56.9%)	123 (61.5%)	104 (52.3%)
Consultant	172 (43.1%)	77 (38.5%)	95 (47.7%)

Complications	All patients	Closed reduction	Open reduction
Nail related complications*	81 (20.1%)	31 (15.4%)	50 (24.9%)
Failure at lag screw junction	20 (5.0%)	6 (3.0%)	14 (7.0%)
Self-dynamisation	20 (5.0%)	8 (4.0%)	12 (6.0%)
Cut-out	10 (2.5%)	5 (2.5%)	5 (2.5%)
Non-union	63 (15.7%)	28 (13.9%)	35 (17.4%)
Peri-implant fracture	12 (3.0%)	4 (2.0%)	8 (4.0%)
Re-operation (nail failure)**	28 (7.0%)	8 (4.0%)	20 (10%)
Re-operation (any cause)***	63 (15.7%)	28 (13.9%)	35 (17.4%)
HAP/CAP	83 (20.6%)	42 (20.9%)	41 (20.4%)
UTI	63 (15.7%)	25 (12.4%)	38 (18.9%)
Wound infection			
Superficial	14 (3.5%)	2 (1.0%)	12 (6.0%)
Deep	10 (2.5%)	2 (1.0%)	8 (4.0%)
CKD Stage pre-operatively			
Mild	273 (69.5%)	144 (73.5%)	129 (65.5%)
Moderate/severe	120 (30.5%)	52 (26.5%)	68 (34.5%)
CKD stage post-operatively			
Mild	285 (72.7%)	156 (79.6%)	129 (65.8%)
Moderate/severe	107 (27.3%)	40 (20.4%)	67 (34.2%)
Pre-operative transfusion	35 (8.7%)	11 (5.5%)	24 (12.0%)
Post-operative transfusion (48 h)	212 (52.7%)	83 (41.5%)	129 (64.5%)
Post-operative transfusion (total)	258 (64.2%)	106 (53.0%)	152 (76.0%)
Hb drop (g/dL)	45.16 (17.92)	48.07 (16.70)	42.29 (18.65)
VTE			
No	78 (84.8%)	36 (85.7%)	42 (84%)
DVT	8 (8.7%)	4 (9.5%)	4 (8%)
PE	6 (6.5%)	2 (4.8%)	4 (8%)

Radiographic measurements	All patients	Closed reduction	Open reduction
Femoral neck shaft angle			
Normal	266 (67.5%)	125 (63.5%)	141 (71.6%)
Coxa Valga	98 (24.9%)	55 (27.9%)	43 (21.8%)
Coxa Vara	30 (7.6%)	17 (8.6%)	13 (6.6%)
Number of fragments (comminution)			
Simple	100 (24.9%)	45 (22.4%)	55 (27.5%)
Moderate	210 (52.4%)	115 (57.2%)	95 (47.5%)
Severe	91 (22.7%)	41 (20.4%)	51 (24.6%)
Isolated subtrochanteric extension	61 (15.2%)	27 (13.4%)	34 (17.0%)
Atypical	16 (3.9%)	9 (4.5%)	7 (3.5%)
Pathological	5 (1.2%)	4 (2.0%)	1 (0.5%)
Distal extension	136 (33.8%)	49 (24.4%)	87 (43.5%)
Lesser trochanter fracture	279 (69.4%)	136 (67.7%)	143 (71.5%)
Medial calcar comminution	25 (6.2%)	16 (8.0%)	9 (4.5%)
Lateral cortex gap size (mm)			
≤ 4	245 (61.4%)	129(64.8%)	116 (58.0%)
5–9	103 (25.8%)	44 (22.1%)	59 (29.5%)
≥ 10	51 (12.8%)	26 (13.1%)	25 (12.5%)
Medial cortex gap size (mm)			
≤ 4	270 (67.7%)	141 (70.9%)	129 (64.5%)
5–9	89 (22.3%)	39 (19.6%)	50 (25.0%)
≥ 10	40 (10.0%)	19 (9.5%)	21 (10.5%)
Anterior cortex gap size (mm)			
≤ 4	265 (66.2%)	129 (64.5%)	136 (68.0%)
5–9	87 (21.8%)	44 (22.0%)	43 (21.5%)
≥ 10	48 (12.0%)	27 (13.5%)	21(10.5%)
Posterior cortex gap size (mm)			
≤ 4	312 (78.0%)	161 (80.5%)	151 (75.5%)
5–9	66 (16.5%)	28 (14.0%)	38 (19.0%)
≥ 10	22 (5.5%)	11 (5.5%)	11 (5.5%)
Reduction angle grouped (degrees)			
Valgus 5–varus 5	290 (72.7%)	149 (74.9%)	141 (70.5%)
Valgus > 5	29 (7.3%)	16 (8.0%)	13 (6.5%)
Varus 5–10	5a9 (14.8%)	25 (12.6%)	34 (17.0%)
Varus > 10	21 (5.2%)	9 (4.5%)	12 (6.0%)
TAD (mm)			
< 25	353 (89.6%)	175 (89.3%)	178 (89.9%)
≥ 25	41 (10.4%)	21 (10.7%)	20 (10.1%)
Distal locking 1	10 (2.5%)	7 (3.5%)	3 (1.5%)
(Number of screws) 2	391 (97.5%)	194 (96.5%)	197 (98.5%)
Method of locking			
Static Locking	263 (66.1%)	132 (66.0%)	131 (66.2%)
Secondary dynamisation	133 (33.4%)	67 (33.5%)	66 (33.3%)
Dynamic	2 (0.5%)	1 (0.5%)	1 (0.5%)
Nail/canal ratio	0.82 (0.08)	0.82 (0.08)	0.82 (0.08)

Hospital stay/mortality	All patients	Closed reduction	Open reduction
HDU/ICU stay	48 (11.9%)	21 (10.4%)	27 (13.4%)
Total length of hospital stay (days)	23.71 (19.25)	23.01 (19.80)	24.42 (18.70)
Weekend admission	134 (33.3%)	68 (33.8%)	66 (32.8%)
Died within a year	72 (17.9%)	29 (14.4%)	43 (21.4%)

Dichotomous variables are presented as absolute numbers (percentages) of the positive event

Continuous variables are presented as mean (SD)

*ISS* injury severity score; *ASA* American society of anaesthesiologists classification; *DEXA* dual-energy X-ray absorptiometry; *FWB* full weight bearing; *PWB* partial weight bearing; *TTWB* toe-touch weight bearing; *NWB* non-weight bearing; *HAP* hospital acquired pneumonia; *CAP* community acquired pneumonia; *UTI* urinary tract infection; *CKD* chronic kidney disease; *DVT* deep vein thrombosis; *VTE* venous thromboembolism; *TAD* tip apex distance; *AP* anterior–posterior view; *LAT* lateral view; *HDU* high dependency unit; *ICU* intensive care unit

^*^Nail related complications: this included nail failure, peri-implant fracture and peri-implant infection

^**^Nail failure: this included failure at lag screw junction, cut-out of the lag screw and breakage of the distal locking screws (self-dynamisation) as nail-failure

^**^^*^Re-operation for all causes included re-operation following nail failure, infection, removal of metalwork for any cause (i.e. impingement, post-traumatic arthritis, removal of distal screws for dynamisation of the nail) and revision for non-union

In the open reduction group, the time to operation was 2.14 ± 2.77 days, duration of operation was 125.22 ± 47.36 min, with a consultant as senior surgeon in 95 cases (47.7%), a length of hospital stay (LOS) of 24.42 ± 18.70 days and reduction angle was neutral (5° valgus to 5° varus) in 70.5% of patients. Within the closed reduction group, the time to operation was 1.83 ± 1.76 days, duration of operation was 96.58 ± 35.60 min, a consultant as senior surgeon in 77 cases (38.5%), LOS was 23.01 ± 19.80 days and reduction angle was neutral (5° valgus to 5° varus) in 74.9% of patients.

When comparing the demographic factors, injury characteristics, comorbidities and operative characteristics of subtrochanteric fractures requiring open reduction against those having a closed reduction, duration of operation [OR: 3.31 (2.15–5.10); *p* < 0.001] and distal fracture extension to diaphysis [OR: 2.39 (1.56–3.66); *p* < 0.001] were statistically different between the two groups (Table 2). Although there was some variation between the open and closed reduction group in terms of Russell-Taylor classification (*p* = 0.146), other than distal fracture extension to diaphysis, we failed to identify any specific fracture patterns associated with a higher risk of an open reduction. No difference in adequacy of reduction was evident comparing the two groups, as demonstrated by the gap size at the fracture site and the reduction angle.

**Table 2:** Unadjusted associations with open reduction

Operation characteristics	Unadjusted OR (95% CI)	*p* value
Surgical time (> 120 min)	3.31 (2.15–5.10)	< 0.001

Complications	Unadjusted OR (95% CI)	*p* value
Nail related complications	1.82 (1.10–2.99)	0.019
Wound infection		
Superficial	6.53 (1.44–29.58)	0.014
Deep	4.35 (0.91–20.77)	0.065
Post-operative transfusion (48 h)	2.56 (1.71–3.84)	< 0.001
Post-operative transfusion (total)	2.81 (1.83–4.30)	< 0.001
Hb drop (g/dL)	0.98 (0.97–0.99)	0.002

Radiographic measurements	Unadjusted OR (95% CI)	*p* value
Distal extension	2.39 (1.56–3.66)	< 0.001

### Complications

Of all complications, only superficial infection, post-operative transfusion and nail-related complications were found to be statistically different between the two groups. Examining the deep infections (including unmatched cases), three were reported in the closed reduction group (two were re-operated) and ten in the open reduction group (eight were re-operated) (Table 3). Nail-related complications {24.9% [open reduction] Vs. 15.4% [closed reduction]; OR: 1.82 (1.10–2.99); *p* = 0.019} and need for transfusion within 48 h post-operatively {64.5% [open reduction] Vs. 41.5% [closed reduction]; OR: 2.56 (1.71–3.84); *p* < 0.001} likewise were higher in the open reduction group. Requirement for pre-operative transfusion, nail failure (breakage at lag screw junction or self-dynamisation), cut-out, fracture non-union, peri-implant fractures, admission to high dependency/intensive care (HDU/ICU) unit, length of hospital stay, 30-day and 1-year mortality rate were similar between the two groups (Table 1). Regarding re-operation risk, we failed to identify any significant difference in re-operation for nail failure (*p* = 0.331) and re-operation for any cause (*p* = 0.410).

**Table 3:** Outcomes of unmatched patients presenting with a deep infection

	Closed reduction	Open reduction	
		No cerclage wiring	Cerclage wiring
Number of pts	3 pts	7 pts	3 pts
Re-operations	2 pts	6 pts	2 pts
Number of re-operations	One pt had 2 and another 5 re-operations	Mean: 6 re-operations Median: 6 re-operations SD: 4.2 re-operations	One pt had 1 and another 3 re-operations
Final outcome (union)	Non-union: 2 pts*	Non-union: 3 pts**	Non-union: 1 pt****
Infection outcome	Remission: 3 pts	Remission: 6 pts***	Remission: 3 pts
Mortality	1 pt	3 pts	1 pt

*pt* patient

*One patient died before union and one patient had a total hip replacement secondary to non-union

**Two patients died before union; one patient had a total hip replacement secondary to non-union; one patient had an asymptomatic infected non-union and managed conservatively

***In one patient the infection lead to multi-organ failure resulting in patient’s death

****Patient died before union

### Factors associated with open reduction and risk of complications

Having adjusted for the different variables associated with open reduction, our logistic regression analysis of the matched groups revealed that open reduction was strongly associated with the following: an increased risk of superficial infection (OR: 7.88; 95% CI 1.63–38.16; *p* = 0.010), a distal extension of the fracture line to the diaphysis (OR: 2.30; 95% CI 1.45–3.65; *p* < 0.001), an increased risk of post-operative transfusion within the first 48 h following surgery (OR: 2.44; 95% CI 1.58–3.76; *p* < 0.001) and a surgical time of greater than 120 min (OR: 3.09; 95% CI 1.96–4.87; *p* < 0.001) (Table 4). Deep wound infection was not significant after correction for covariates (*p* = 0.160).

**Table 4:** Multivariate models demonstrating associations of open reduction following a subtrochanteric fracture

	OR	95% CI	Pr ( >|z|)
Wound infection (superficial)	7.88	1.63–38.16	0.010
Wound infection (deep)	3.21	0.63–16.33	0.160
Surgical time (> 120 min)	3.09	1.96–4.87	< 0.001
Transfusion within 48 h post-operatively	2.44	1.58–3.76	< 0.001
Distal extension	2.30	1.45–3.65	< 0.001

*OR* odds ratio, *CI* confidence interval

### Subgroup analysis: ‘clamp assisted only’ vs. ‘cerclage wiring’ open reduction

A further subgroup regression analysis (Table 5) of all matched fractures requiring open reduction (matched against age, gender and mechanism of injury) revealed that open reduction using cerclage wiring was more commonly necessitated when there was a distal fracture extension (OR: 10.23; 95% CI 4.81–21.78; *p* < 0.001). Although open reduction with cerclage wiring was associated with a prolonged surgical time (OR 6.43; 95% CI 3.12–13.26; *p* < 0.001), it was associated with a smaller risk of developing a non-union when compared to ‘clamp assisted only’ open reduction (OR: 0.20; 95% CI 0.06–0.74; *p* = 0.015).

**Table 5:** Multivariate models demonstrating associations of ‘clamp assisted only’ open reduction vs. open reduction using cerclage wire/cable following a subtrochanteric fracture

	OR	95% CI	Pr ( >|z|)
Distal extension	10.23	4.81–21.78	< 0.001
Surgical time > 120 min	6.43	3.12–13.26	< 0.001
Non-union	0.20	0.06–0.74	0.015

*OR* odds ratio, *CI* confidence interval

## Discussion

Subtrochanteric fractures have been previously associated with a high risk of complications, some of which could be potentially preventable [22–24]. In the literature, there is still a lack of evidence comparing the outcomes of open versus closed reduction [22, 25–28]. Most of these studies are small in sample size [22, 25, 27], whilst some assess the elderly age group only [27], but no study reports on the associations of open reduction. Our study, therefore, represents the first study reporting on the associations of open reduction in subtrochanteric femur fractures and related complications.

Open reduction has traditionally been met with some reluctance. This is due to the misbelief that open reduction may compromise the local fracture biology and vascularity [5], thereby reducing the threshold of what could be deemed as an ‘acceptable’ suboptimal reduction. Similarly, cerclage wiring was believed to cause strangulation of the bone, risking necrosis and ultimately failure of healing [29]. Recent studies have, however,, challenged this school of thought. Histological and anatomical studies by Nather et al. [30] and Pazzaglia et al. [31] have both described the periosteal blood supply as circumferential. Lenz et al.’s ex-vivo study using human diaphyseal bone further confirmed this, whereby they found no periosteal compression when using a cerclage wire or a cable [32, 33]. Additionally, in a systematic review by Förch et al. on the effect on cerclage wiring to periosteal perfusion, the group reported no significant evidence to support this [34]. Furthermore, biomechanical studies have also demonstrated how the use of cerclage wire is advantageous, as it stabilises the medial hinge and raises the threshold of cyclical compressive loading tolerated by the femur before reaching plastic deformation [35]—factors found to significantly decrease the rate of implant failure following IM nailing [35].

Contrary to the bimodal age and gender distribution [2], and unimodal older male and female distribution reported in the literature [36–38], we identified a bimodal male, unimodal older female age distribution in our cohort. The majority of subtrochanteric fractures (82.4%) in our adult population had comminution or distal diaphyseal extension—all characteristics of an unstable fracture pattern. This, therefore, explains the high rate of open reduction demonstrated in our study population (48%), which is in keeping with that reported by other studies (range: 33.3–82.2%) [25–27]. To reduce the bias of our outcomes, we matched the two groups as per age, gender and mechanism of injury. Only distal fracture extension to the diaphysis was found to be different (*p* < 0.001), whilst the rest of the fracture characteristics were comparable in the two groups. Furthermore, our regression analysis confirmed that open reduction was associated with a longer surgical time (OR: 3.09), which suggests a more challenging fracture reduction mandating open reduction in the first place.

Kilinc et al. reported a 5.8% superficial and 3.9% of deep infection in their study on 52 patients with cerclage wiring of subtrochanteric fractures treated with IM nailing [39]. Studies comparing open and closed reduction of subtrochanteric fractures treated with cephalomedullary nailing reported similar infection rates between the two groups, which range between 0 and 1.7% [25–28, 40]. These studies, however, are limited by their small sample size and restricted age groups. Having overcome the aforementioned limitations, our study has conclusively revealed that there is a statistically significant higher risk of developing superficial wound infection in the open reduction group (OR: 7.88). Contrary to common belief, deep infection was not statistically increased amongst the open reduction group.

There is a paucity of studies in the literature which compared the blood transfusion requirement between subtrochanteric fractures requiring open versus closed reduction. Codesido et al. reported no difference in transfusion requirements between the two groups [26], whereas Shukla et al. reported higher transfusion requirement in the open reduction group [27]. We found that open reduction poses a higher risk of requiring blood transfusion within the initial 48-h postoperative period (OR: 2.44). The higher transfusion risk could be explained by our findings wherein open reduction was performed in fractures requiring a prolonged surgical time, more extensive surgical dissection and, therefore, soft tissue damage—all contributory factors towards a higher bleeding risk.

Collectively, we found that open reduction was associated with a higher risk of nail complications than closed reduction (*p* = 0.031). However, this difference was no longer significant when we individually assessed each of the nail complications. The small sample size for each of these complications and, therefore, an underpowered comparison (Type II error) most likely accounts for the lack of statistical difference.

With regard to the non-union rates, these have been reported to be lower in open reduction group [25, 26]. Karayiannis et al. reported rates of symptomatic non-union to be 5.8% and 6.3% in the open reduction and closed reduction group, respectively [25]. Codesido reported no non-unions amongst subtrochanteric fractures requiring open reduction, whilst 8.3% of the closed reduction group developed non-union [26]. Kilinc et al. (cerclage wire reduction) and Mingo-Robinet et al. (clamp assisted reduction only) both reported no non-unions in their cohorts of patients [28, 39]. Out of the three main operative factors, Krappinger et al. found that the lack of medial cortical support and varus malalignment as significant risk factors for non-union in subtrochanteric fractures treated by IM nailing [3]. Even though our study has demonstrated no difference in the non-union rates between subtrochanteric fractures treated with closed or open reduction, we did identify that open anatomical reduction achieved by cerclage wire/cable to have a lower non-union rate (OR: 0.20), when compared to ‘clamp assisted only’ open reduction. One could assume that higher non-union rates seen in open reduction group were secondary to suboptimal exposure and reduction. Interestingly, the use of cerclage wires/cables seems to reduce the risk of non-union. Possible mechanisms behind this could be a better reduction of the fracture with smaller fracture gaps and restoration of the medial buttress.

To our knowledge, our study is the largest cohort series reported in the literature to date comparing the outcomes of closed versus open reduction of subtrochanteric femur fractures treated with IM nailing. With no exclusion criteria posed upon age or comorbidity, our study provides a better overview of the overall fracture demographics and epidemiology of subtrochanteric femur fractures encountered in a Level 1 Trauma Centre serving a metropolitan population. By matching the two groups with respect to age, gender and mechanism of injury, we reduced bias that could result from baseline differences between the two populations. The retrospective nature of our study on the other hand meant certain data may be subjected to bias. Although redness, swelling and mild discharge in the first few post-operative days are signs of physiological inflammation of wound healing, this could easily be clinically over-diagnosed as ‘superficial wound infection’ by non-orthopaedic physicians, junior doctors, district nurses or advance nursing practitioners who are less experienced in assessing surgical wounds. Therefore, we may have overestimated this risk, with an inherent bias of superficial infection towards open reduction. We also accept that the assessment of fracture pattern is subject to intra- and inter-observer reliability. We did, however, attempt to overcome this weakness by having two independent assessors for the analysis of fracture pattern and radiological measurements. Of note, fractures requiring an open reduction are generally more difficult to reduce and, therefore, are subjected to a higher risk of complications including non-union, hence a potential bias against this group.

### Conclusion

Open reduction of subtrochanteric fractures is not associated with an increased risk of deep infection and non-union. It is, however, associated with an increased risk of superficial infection, transfusion within 48 h following surgery and a prolonged surgical time. Furthermore, we found that open anatomical reduction achieved by cerclage wiring to have a lower non-union rate when compared to ‘clamp assisted only’ open reduction. Optimal anatomical fracture reduction of subtrochanteric femur fractures during primary surgery should, therefore, be prioritised, even if an open reduction is required. We do not, however, advocate open reduction of fractures where adequate reduction can be achieved by closed means, as this could be linked to an unnecessary risk of complications.

## Data Availability

The data that support the findings of this study are available from the corresponding author, MP, upon reasonable request.
